# Supplementary material for: TrackStudio: An integrated toolkit for markerless tracking
Source: PLoS One. 2026 Sep 23;21(9):e0358647. doi: 10.1371/journal.pone.0358647 (PMC13600507; doi:10.1371/journal.pone.0358647)
Supplement: Supplementary file 1 [file pone.0358647.s001.pdf]

S1 Appendix – TrackStudio Manual

Prerequisites

For installation purposes, the user should already have Anaconda software installed (https://www.anaconda.com/docs/getting-started/anaconda/install). Before installing the TrackStudio toolkit, download the toolkit's files from GitHub (https://github.com/dimitrov-hristo/TrackStudio) and virtual environments files from FigShare (https://doi.org/10.6084/m9.figshare.30556418.v1). The unzipped toolkit files and the 2 virtual environment .tar files must all be in the same folder (see S1 Fig).

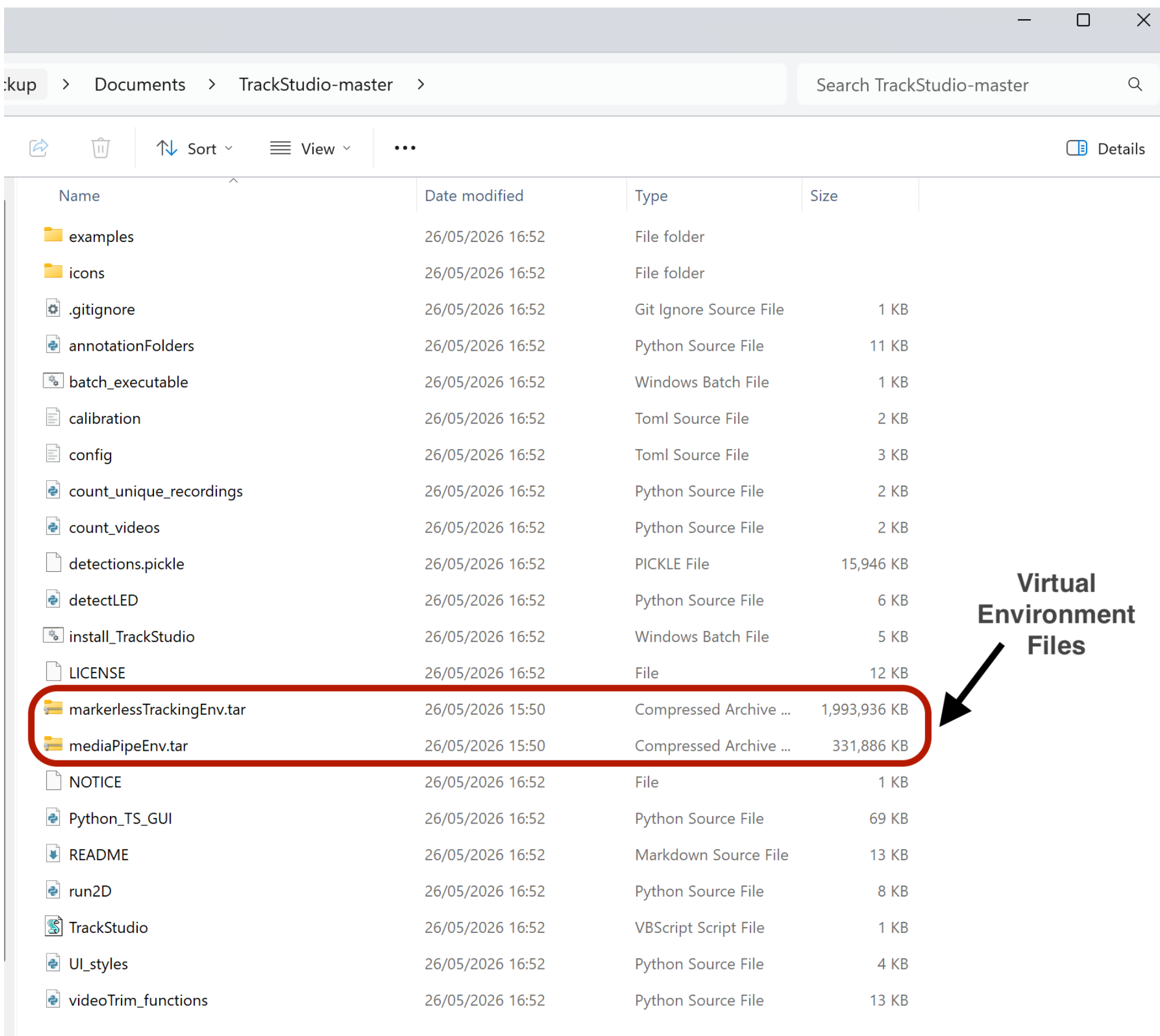

S1 Fig. TrackStudio master folder before installation.

Installation

To install TrackStudio you need to double-click the installation file (install_TrackStudio.bat). The installation process may take 10-40 minutes. In the unlikely event the installer cannot automatically find your Anaconda folder, you will be prompted to enter the path. During installation (S2 Fig), the installer will:

1. Set up MediaPipe virtual environment → prompts "press any key" to continue.

2. Set up Anipose virtual environment (this is the longest step) and run a quick installation test → prompts “press any key.”

3. Update TrackStudio files → prompts “press any key.”

4. Finalise the setup → final “press any key,” and you are done.

5. Open the TrackStudio GUI by double-clicking the ’TrackStudio.vbs’ file.

Note: The first launch of the GUI (after a double-click of the ’TrackStudio.vbs’ file) may take extra time while initial variables are created – wait until the GUI has been fully initiated before trying anything else. If the GUI does not start after 20-30s, try opening the Anaconda software as well as opening the ’batch_executable.bat’ and the ’TrackStudio.vbs’ files as text files (e.g. in Notepad) to check if the paths within the ’batch_executable.bat’ and the ’TrackStudio.vbs’ files have been updated correctly.

These paths should have been updated automatically. However, in case of an issue with the process:

- The path in the second line of ’TrackStudio.vbs’ should have been updated from the default `D:\Markerless_Tracking_GUI_git\batch_executable.bat` to the path of the folder where the track studio GitHub repository has been unzipped.

- Similarly, the path in the last line of the ’batch_executable.bat’ should point towards the location of the ’Python_TS_GUI.py’ file in your computer (where you’ve unzipped the GitHub repository) rather than the default `D:\Markerless_Tracking_GUI_git\Python_TS_GUI.py` and the path to the batch file for activating Anaconda in the third line of the ’batch_executable.bat’ should point towards the one within your Anaconda installation.

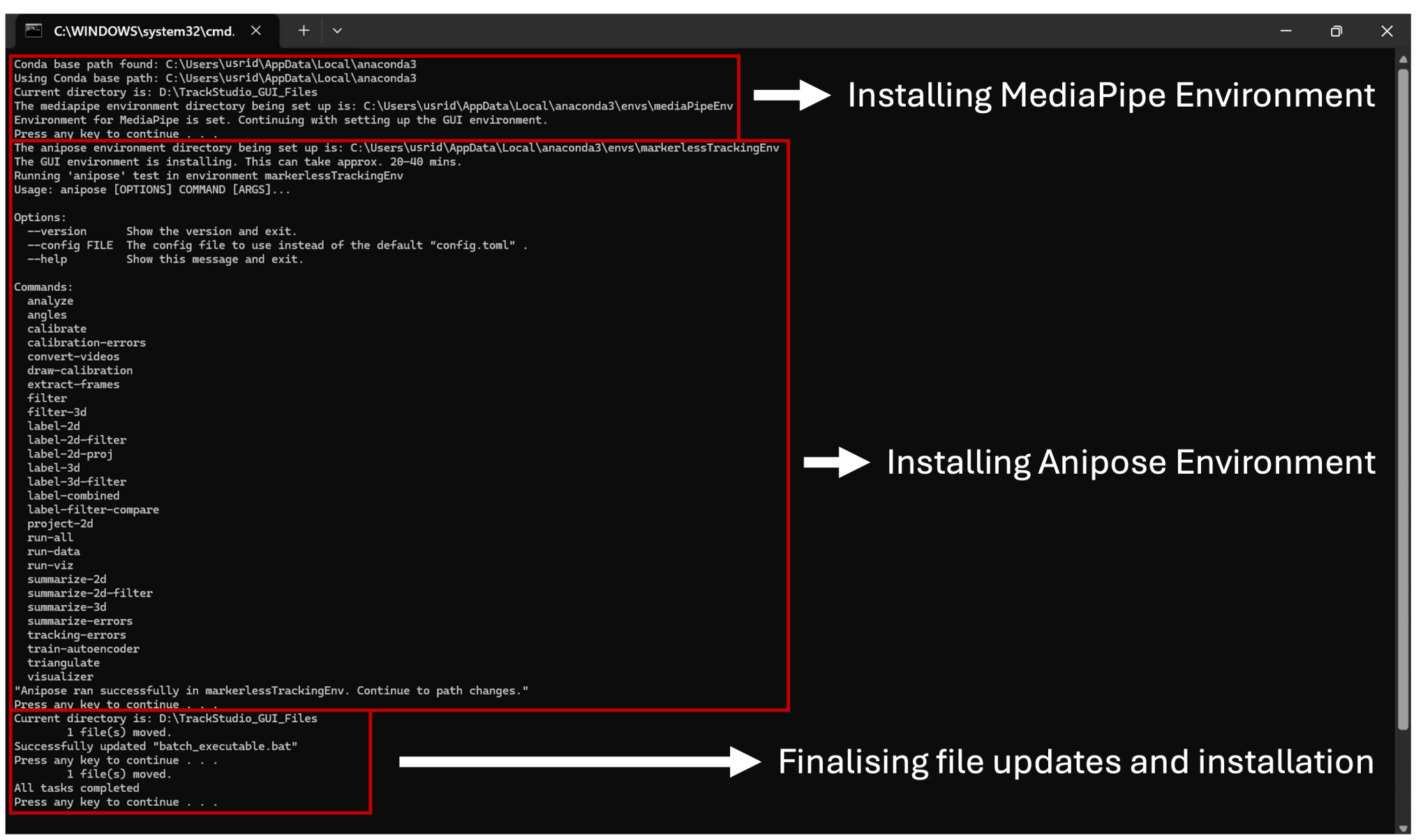

S2 Fig. Installation window visualisation, indicating where each step takes place.

After installation, the TrackStudio GUI can be launched by double-clicking the application file (TrackStudio.vbs). Advanced code information is provided on the GitHub page (https://github.com/dimitrov-hristo/TrackStudio).

There is an ‘examples’ folder provided for people to go through examples using the instructions from the ‘Usage’ section below and familiarise themselves with the GUI.

The examples are located in `TrackStudio/examples/videos` within the GitHub page. They contain **.mp4** recordings from 3 web cameras with file naming suffix '**-cam([A-Z])**' of a person performing an action with their **right hand**. People can try the trimming functionality of the GUI by trimming videos by an LED light approach using the 'raw-videos' folder, or performing 2D annotation using the 'trimmed-videos' folder and subsequently performing camera calibration and 3D annotation using the 'calibration' folder and the subsequently generated files.

Before You Start: Recording & Organising Video Recordings

Video Recordings

An easy way to record several cameras on one computer is to use OBS (Open Broadcaster Software, Lain Bailey, Michigan, US) [1]. Open one OBS window per camera (e.g. three cameras → three OBS windows). Example OBS profiles and settings are available on the project's GitHub.

Naming & Saving Camera Recordings

For a seamless MLT process, always save your video footage in a dedicated directory for video recordings. TrackStudio will mirror your folders (e.g. participant → task → trial), but the lowest-level folder (e.g. trial1) must contain video files only—no notes, images, or other files. When recording cameras, give each camera a unique name using this format:

- Start with '_' or '-'
- Then 'cam'
- Then a lowercase/capital letter (a-z)/(A-Z) or number (1-9)

3-camera examples: '-camA, -camB, -camC' or '_cam1, _cam2, _cam3'. You can set the suffixes used for each OBS window in Advanced Settings → Recording and File Formatting (see S3 Fig).

<u>Optional: Using more than 3 webcams</u>

Although most users will not need this, if using more than three cameras or high-resolution/high-frame-rate cameras, please check:

- Cables/ports: Use cables that support your camera needs (e.g. high-resolution cameras may require USB-C 3.2 Gen 2 or better). Slow cables can cause dropped frames.
- USB port limits: A laptop/desktop might have four USB-C ports but only two internal USB controllers ('boards'), meaning the connections are shared. If video lengths are not matching due to USB speed limitations, add a dedicated USB expansion card (desktop) or reduce the resolution/frame rate per camera.
- Computer performance: Your computer must be able to process and display multiple live video streams and save them to its hard drive. If it cannot, video frames may skip at random (these cannot be recovered). *Tips*: Record to a fast SSD, close other apps, reduce previews, and consider lowering FPS/resolution.

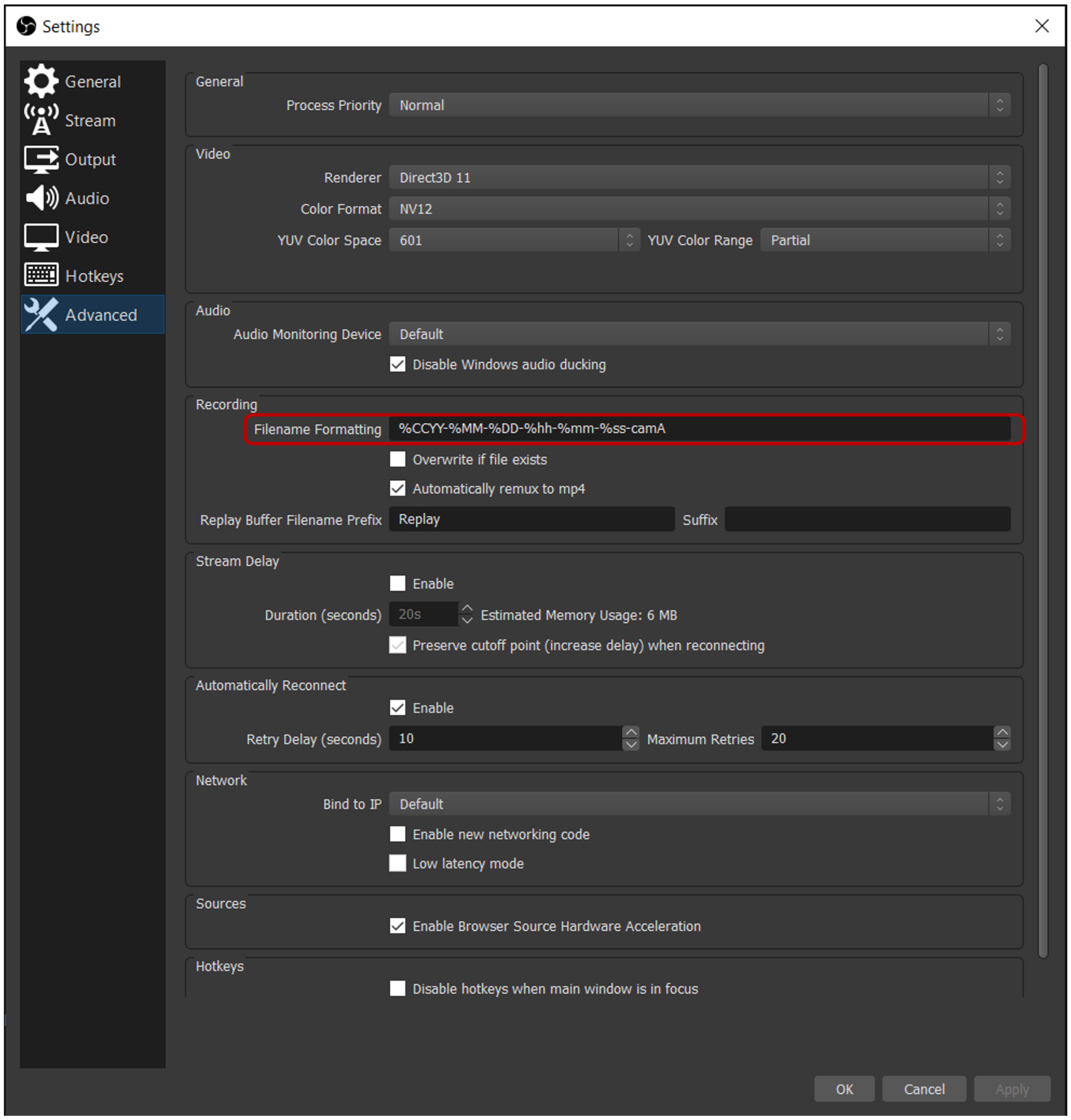

S3 Fig. Setting section of Open Broadcasting Software (OBS). Each camera recording should have a unique suffix at the end of the recorded file name.

Synchronisation

There are two different kinds of synchronisation:

1. Event synchronisation – aligning videos with your task events (e.g. trial start/end).
2. Camera synchronisation – making sure all cameras show the same moment on the same video frame.

For 3D MLT, camera synchronisation is **essential**. Even a small delay (tens of milliseconds) can break the 3D estimation and corrupt the obtained results. For example, camera A might display an opened hand, while camera B already shows the hand closing. If you build a 3D view from those two video frames, the result will be corrupted as the two views are from different moments in time.

Important note about global computer timestamps

Global computer timestamps are useful for event synchronisation only (matching videos to your task). They do not make cameras aligned with each other. Each camera video is independent when it transfers the data to the computer. Hence, a simple start/stop timestamps can leave cameras out of synchronisation. Some softwares can extract timestamps for each camera separately, for example, LabRecorder (https://github.com/labstreaminglayer/App-LabRecorder) [2] (see below).

<u>Recommended ways to synchronise cameras</u>

1. Visual or audio cue (**recommended**): Use an LED light that turns ON/OFF at known times (e.g. task start/transition/end) and is visible in every camera or use a sharp sound (a beep) recorded by all cameras. This provides a clear, shared timing marker you can see or hear later and verify it. If using sound, ensure audio is recorded in the video files.

2. Software timestamping: Use of a software tool, such as LabRecorder [2], to extract separate timestamps per camera and event. Often adequate for slower motions or tolerant tasks, but can be inaccurate for time-critical applications, and cannot be verified.

<u>Best practices for LED light synchronisation</u>

1. Make sure every camera can see the LED clearly at all times. If not, add more LEDs that switch ON/OFF together. *Tips*: for multiple LEDs, you can place an LED in the corner of each camera, which does not block the main view but is always visible.

2. Use a dark background behind the LED (e.g. black cloth) that the ON/OFF changes are obvious.

3. Do not let anything block the LED during ON/OFF events. If it is hidden in any view, synchronising and auto-trimming may fail. If a body part occludes the LED light, the video trimming tool may incorrectly interpret the skin tone as a change in LED colour (particularly when using a red LED). This may lead to the misidentification of ON/OFF events and result in inaccurate video trimming

4. Simple LED wiring and an Arduino script example are available on GitHub (https://github.com/dimitrov-hristo/TrackStudio).

Quick start checklist

- [ ] One OBS window per camera; you can use the provided OBS settings.
- [ ] Name cameras `-camA`, `-camB`, `-camC` or `_cam1`, `_cam2`, ...
- [ ] If using 4+ cameras: check cable speed, USB bandwidth/limits, and computer performance; a fast SSD helps.
- [ ] Choose a synchronisation method if required:
  - For 2D task alignment only → global computer timestamps; this does not align cameras.
  - Basic camera synchronisation → software timestamping.
  - Preferred camera sync → LED or sound that is visible/audible to every camera.
- [ ] Record a 10–15 s test. Check for no dropped frames and verify the sync cue occurs on the same frame number in every view.

Record a 1-minute test. Check for no dropped frames and that the synchronisation cue appears in every view (sample script available on GitHub - https://github.com/dimitrov-hristo/TrackStudio).

Usage

TrackStudio (S4 Fig) is operated through a graphical interface, but it does not enforce a step-by-step wizard. Hence, complete tasks in order according to your goal:

- If you need 3D Annotation, you must first run 2D Annotation AND Camera Calibration. Make sure that camera views are time-aligned (see Synchronisation above).
- If you need to label/export 2D and/or 3D videos, you must first generate the 2D and/or 3D Annotations for them.

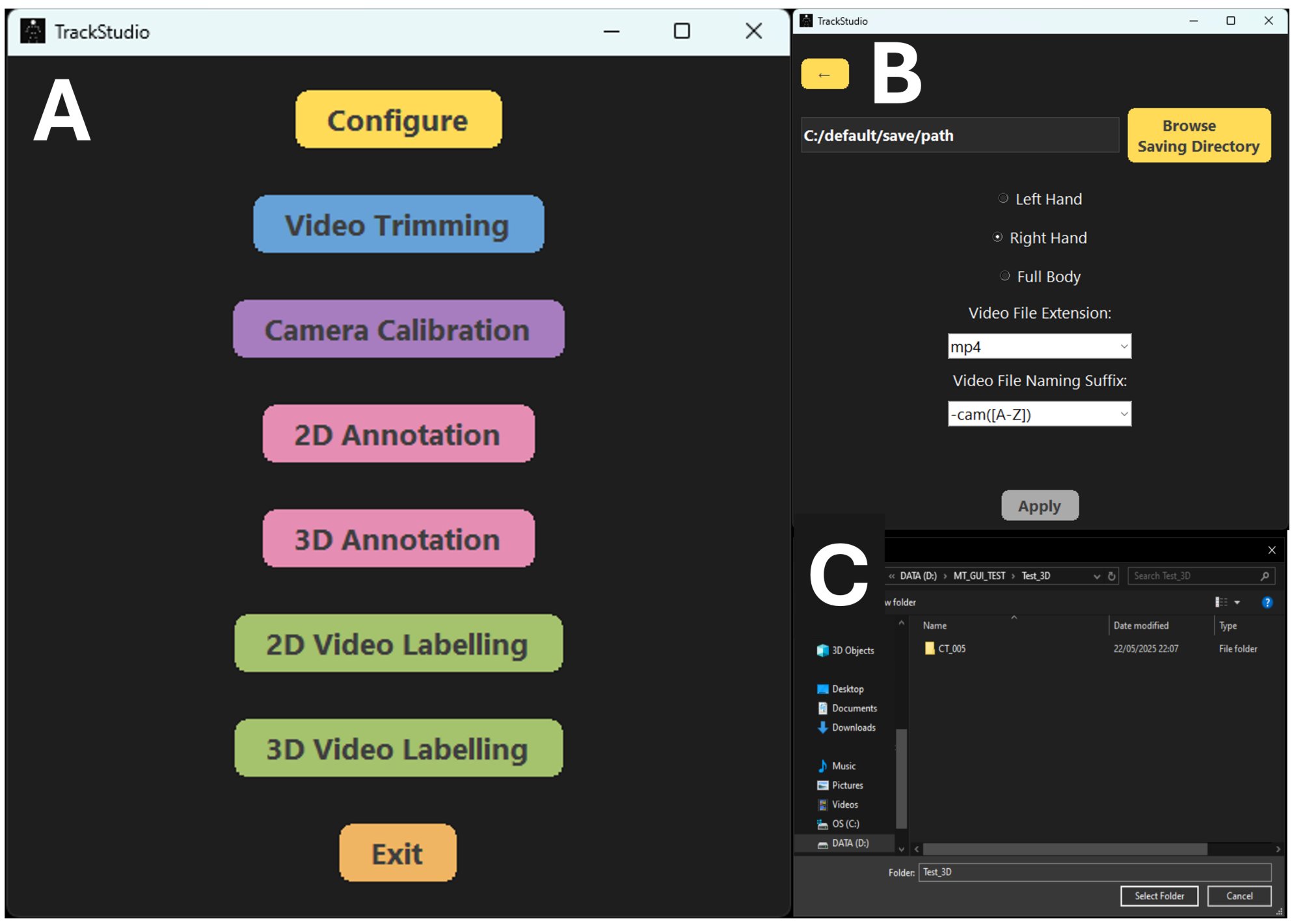

S4 Fig. TrackStudio GUI. A: TrackStudio main window. B: Configuration section of TrackStudio GUI. C: Example of prompt to select input directory (e.g. for Automatic Video Trimming, 2D/3D Annotation, or 2D/3D Video Labelling).

Selecting Saving Directory for TrackStudio

TrackStudio is designed to handle any folder structure you have for your data, as long as you have **video recordings only** in the final folder (e.g. 'Task 1', see S4 Fig). When you choose a 'Saving Directory' in the GUI, there can be two instances:

1. **First time using this folder – new Saving Directory** (S5 Fig A)
   - If you have never run TrackStudio in the selected directory, the first processing step you run (e.g. Video Trimming or 2D Annotation) will:

    - Recreate your original folder structure (e.g. participant → task → trial) inside the Saving Directory, and
    - Saves trimmed videos there (if Video Trimming is first selected) or copies your video files into the directory (if 2D Annotation is selected).
  - Your original files stay where they are (S5 Fig A – 'Original Recording Directory'). TrackStudio works from the trimmed videos or copies (S5 Fig A – 'New Empty Saving Directory').

2. **Continuing in an existing MLT folder** (S5 Fig B)
   - This folder already has results from Video Trimming and/or 2D Annotation and later steps.
   - TrackStudio will use it as your working folder and save any new results alongside the existing ones.
   - No new directories are created.

Tips:

- Choose a fast drive (SSD) for your Saving Directory to reduce processing time.
- Make sure there is enough free space (videos are copied the first time) – TrackStudio will notify you if larger files are moved.
- Keep the lowest-level folders video-only to avoid import problems.

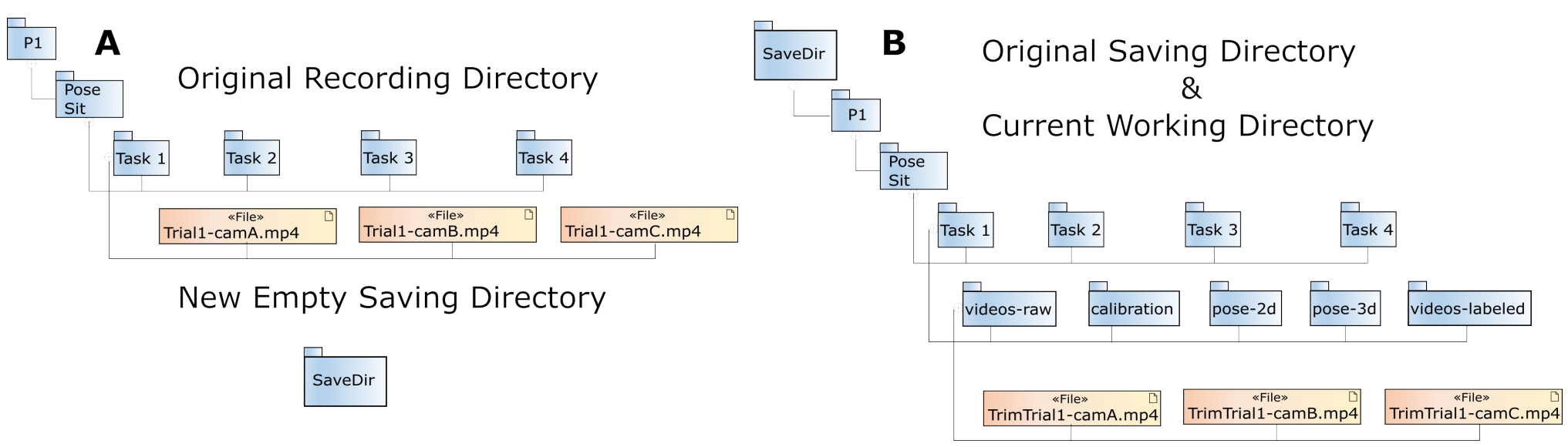

S5 Fig. Examples of the two cases for selecting a 'Saving Directory'. A: An example of the beginning of the MLT process, where no steps have been executed and an empty folder is chosen as the 'Saving Directory'. B: An example of a 'Saving Directory' where some MLT work (calibration, 2D & 3D annotation, and 2D video labelling) has already been done. If TrackStudio is re-opened and the user wants to complete, for example, 3D video labelling for all of the files that they have previously worked on, they should choose this 'Working Directory' as a 'Saving Directory'.

Steps for Markerless Tracking with TrackStudio

TrackStudio includes several tools ("windows"). You only use the ones you need, but some steps depend on others:

- 2D Video Labelling requires 2D Annotation first.
- 3D Annotation requires Camera Calibration and 2D Annotation.
- 3D Video Labelling comes after 3D Annotation.

- 2D/3D Video Labelling is optional - skip it if you do not need labelled videos for visualisation.

You can stop at any time and resume later; your progress is saved in your Saving Directory. For instance, after completing the Camera Calibration, you can close the GUI and proceed with 3D Annotation next time you open TrackStudio, by selecting the same Saving Directory.

When TrackStudio first opens, only Configure and Video Trimming are enabled. Complete the Configure step to unlock the other options.

Below are all the steps of MLT using TrackStudio:

1. **(Always Necessary on Launch!) Configure** – every time you open the GUI, you need to complete a configuration step (see S4 Fig A and B), consisting of selecting:

   (a) **Saving Directory -** location where TrackStudio will store all your results (see 'Selecting Saving Directory for TrackStudio' above).

   (b) **Body part to track** – Right Hand, Left Hand, or Full Body. If you want to track multiple body parts, then you will need to go through all the MLT steps for each body part separately.

   (c) **Video file extension** – e.g. .mp4 or .mkv (match what you have recorded originally for your video files).

   (d) **Camera name suffix** – the text at the end of the recording name that identifies each camera file. For example, if you have 3 cameras and your Trial1 video files are named 'Trial1–camA.mp4', 'Trial1–camB.mp4', and 'Trial1–camC.mp4', then the file naming suffix is going to be '–cam([A-Z])'.

   Once the configuration is completed click 'Apply' and go back to the Start Menu.

2. **Video Trimming** – use this if you need to trim your videos and/or synchronise them using an LED(s) light. When you click Video Trimming, choose a mode:

   (a) **Manual**

   - Pick a video, set start and end frames (use the arrow controls or type a number), then click **Trim**.
   - The trimmed video is saved under videos-raw, inside your Saving Directory (TrackStudio mirrors your original folder structure).
   - Repeat for the next video.

   (b) **Automatic (lights) -** see 'Best practices for LED synchronisation' above

   - Based on detecting a red LED light turning ON at the start and OFF at the end of a trial/event.
   - Choose a folder (a participant folder, a trial folder, or anything in between). TrackStudio will scan and trim all videos in subfolders.
   - Click Select ROI (Region of Interest) – see S6 Fig. For each camera view:
     - A frame appears. Draw a small square around the LED.
     - Press Enter to move to the next camera view and repeat.
     - When all cameras are marked, you will see 'ROI Selected' – click **Trim**.
   - Trimmed videos are saved under "videos-raw" folder in your Saving Directory.

Advanced options for Automatic (lights) video trimming

- Multiple trims within a video – set how many ON–OFF trials/events are inside one long recording. It will trim that number of videos.
- Recording length (in seconds) – enter a fixed duration if every trial/event has the same length (improves cut accuracy).
- Light On threshold (0–255) – set pixel light intensity threshold. Increase if your LED is dim; 255 is pure bright red.
- Pixel number threshold – set how many pixels need to go above the set light on threshold. Typically between 1 and 10 – higher means less false positives but also less sensitive to light changes.

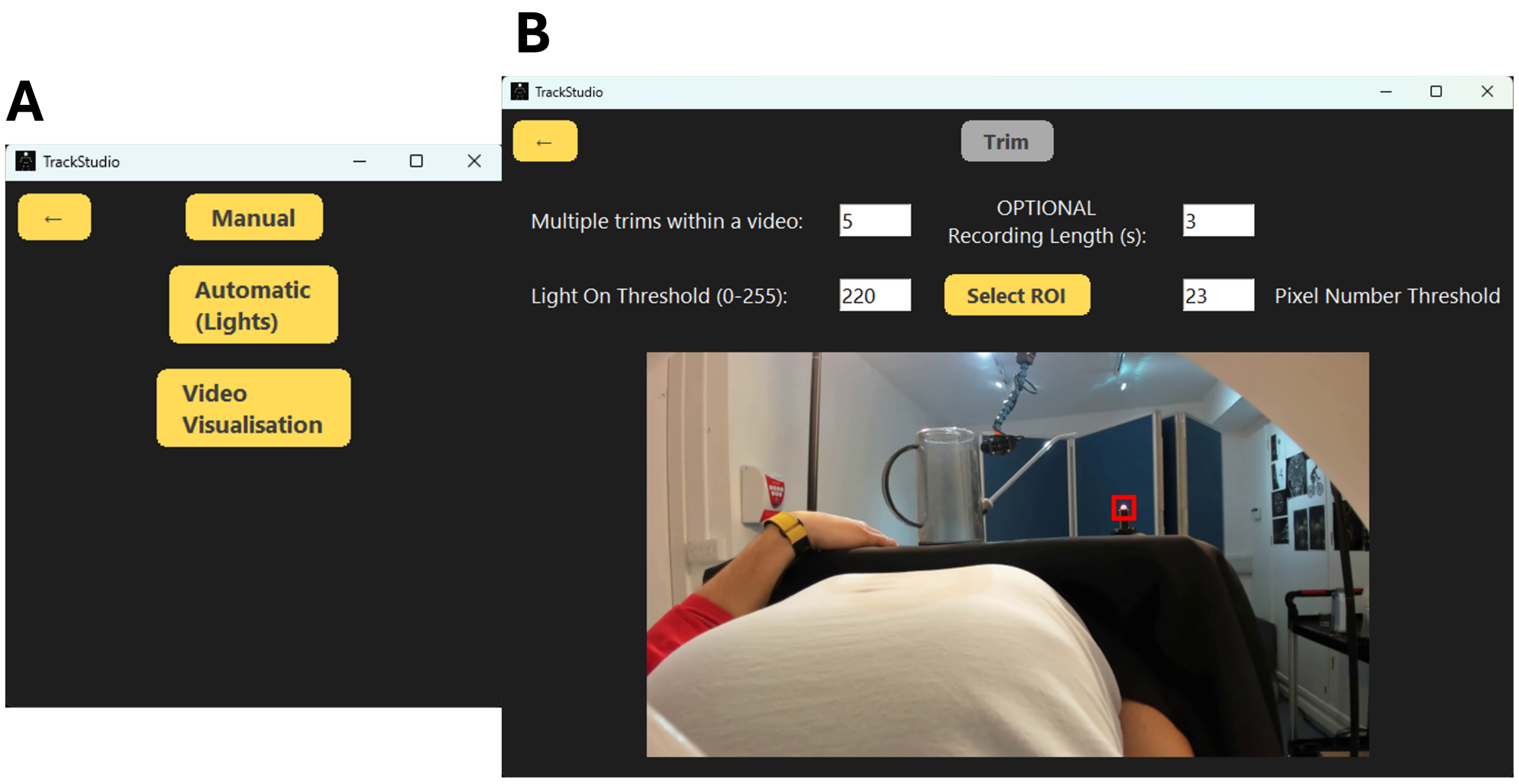

S6 Fig. TrakStudio's Video Trimming. A: Video Trimming window, with Automatic (Lights) window, Manual, and Visualisation. B: Automatic trimming, illustrating LED region selection and optional trimming settings.

3. **Camera Calibration (required for 3D)** – the Camera Calibration window, contains 2 sub-windows '**Change Board Parameters**' and '**Calibrate**':

   (a) **Change Board Parameters** – select only if you used a non-default calibration board (size, type, etc. – see Anipose documentation for details: https://anipose.readthedocs.io/en/latest/params.html). It will allow you to specify your calibration board parameters in a graphical way (no coding needed).

   (b) **Calibrate** – select the folder with your calibration videos. TrackStudio will estimate each camera's distortion and alignment parameters. During calibration:

   - GUI window will display calibration progress messages.
   - Once complete, final calibration error will be displayed
   - A calibration file (calibration.toml) containing camera parameters is saved in a calibration/ folder inside your Saving Directory (and updated in TrackStudio). your saving directory as well as update this file in your TrackStudio directory.

<u>**IMPORTANT**</u>

- Do this before 3D Annotation.
- Quality matters: poor calibration footage can cause calibration to fail or result in highly inaccurate 3D Annotation. The Calibration Practical Recommendation section provides guidance on achieving reliable, high-quality calibration recordings.
- If you record multiple calibrations per participant (e.g. seated vs standing camera positions), you have to work in the sub-directory of each of the calibrations for 'Calibration' and the following '3D Annotation' and later steps for that participant. For example, run Standing calibration → 3D Annotation (standing tasks) before you do the Seated calibration. If you do Standing calibration → Seated calibration → 3D Annotation (standing tasks), TrackStudio will use the Seated calibration and the standing 3D results will be wrong.

4. **2D Annotation** – this step performs 2D MLT and creates files containing 2D coordinates of virtual markers corresponding to the body part you have chosen in the configuration section.
   - Select a folder that contains your trimmed (and synchronised if 3D required later on) videos.
   - Outputs are saved in your Saving Directory, mirroring your original structure and adding the needed subfolders for the underlying open-source tools.
5. **3D Annotation** – combines 2D Annotation results across cameras (using your calibration) to produce 3D coordinates.
   - Requirements: 2D Annotation and a matching Calibration completed for the selected folder directory.
   - Select a folder (within your Saving Directory) that shares one calibration. For example:
     - If Participant_001 used one calibration for all tasks, select the Participant_001 folder for calibration and 3D Annotation.
     - If Participant_001 used multiple calibrations within a session (e.g. seated vs standing), run Calibration and 3D Annotation per task within the task's folder (e.g. `Participant_001/Seated/`).
6. **2D Video Labelling** (optional) – overlays 2D Annotation results onto their respective videos, visualising the results of the tracked body parts.
   - Select the folder (within your Saving Directory) that contains the 2D Annotation results for the videos you want to review.
   - Useful for quality checks; not required for 3D Annotation.
7. **3D Video Labelling** (optional) – creates a tiled video showing the original views with overlayed 3D Annotation results for visualisation.
   - Select the folder (within your Saving Directory) that contains the 3D Annotation results for the videos you want to review.
   - Note: if a recording used more than 4 cameras, the combined preview shows overlays on the first 4 views only (a limitation of the underlying video tiling library). Your 3D data is still computed from all the cameras you used.

For advanced users and developers, the GitHub page explains how each script works and how to adjust or extend TrackStudio (e.g. to swap in another 2D/3D tracking method).

Practical Recommendations

Visibility & Camera Views

- Minimise occlusions whenever possible.
- Do a short test, recording complete task movements, and decide how many cameras are needed to fully capture all movements in the space.
- For 3D MLT, the tracked body part must be visible in at least two camera views at all times. Hence, at least 2 cameras are needed but it is recommended to use 3 or more.
- Full-body or complex movements will require a higher number of cameras and a larger space for recording, with cameras evenly distributed around the room and positioned at different heights.

Frame Rate & Hardware

- For rapid movements, high frame rate cameras (120–240 fps) are advisable to reduce motion blur and maintain stable tracking.
- More/higher-end cameras increase load: you will need fast USB 3.x ports, a strong CPU, and often a GPU for smooth preview (see 'Video Recordings').
- If the system cannot keep up, you will get dropped frames (lost data).

Lighting & Clothing

- Keep the space well-lit and avoid shadows, especially during calibration.
- Avoid loose clothing or patterned fabrics – they may interfere with body landmark detection, similar to challenges in optical tracking.
- Prefer simple, well-fitting clothes (e.g. scrubs), especially for full-body recordings

Recommendations for Improving Calibration Video Recordings:

1. Ensure the environment is well-lit. Poor lighting can prevent the calibration board from being properly detected.
2. Avoid reflections on the printed calibration board. Glossy paper, clear tape, or laminating the board may introduce reflections that interfere with detection.
3. Verify the calibration board is printed at the correct scale and with high contrast. Some printers automatically resize prints to fit the page, which may compromise calibration accuracy. The dark areas of the board should be fully black to enhance feature detection.
4. Leave sufficient margins for holding the board. This prevents hand occlusions. Mounting a handle at the back of the board is recommended.
5. Use a ChArUco calibration board. This type is more tolerant to occlusions and partial views. Not all board corners must be visible in every camera view.
6. We recommend recording calibration videos for at least 1 minute per camera. For example, with three cameras, a total calibration duration of approximately 3 minutes is advised.

7. Monitor the recording during calibration. Position the board so that all cameras have an unobstructed view. This should be your starting and ending position.
   (a) Begin recording and keep the board visible to all cameras for approximately 20 seconds.
   (b) Then, move the board closer to one camera at a time. Each up-close view should be visible to only one camera at a time (avoid partial board views in neighbouring cameras).
   (c) During the up-close phase, hold the board steady, then gently rotate it to capture different angles and lighting conditions. Spend about 40 seconds to 1 minute per camera.
   (d) After each close-up, slowly move the board backwards until it is fully visible in two cameras. Hold it in this position briefly and rotate it slightly to capture various perspectives (while avoiding partial detections).
   (e) Repeat the process for each camera so that all cameras record both up-close and shared views (e.g., A+B, B+C, A+C).
   (f) Finally, return to the starting position where all cameras can see the full board, and slowly rotate it again.

References

1. Bailey H. OBS Project Contributors; 2017. Available from: https://www.obsproject.org/.
2. Kothe C, Shirazi SY, Stenner T, David CM, Boulay, Grivich MI, et al. The Lab Streaming Layer for Synchronized Multimodal Recording. Imaging Neuroscience. 2025;3:IMAG.a.136. Open Access. Available from: https://doi.org/10.1162/IMAG.a.136. doi:10.1162/IMAG.a.136.
